# Development of a Sensitive Colorimetric Indicator for Detecting Beef Spoilage in Smart Packaging

**DOI:** 10.3390/s24123939

**Published:** 2024-06-18

**Authors:** Dariush Karimi Alavijeh, Bentolhoda Heli, Abdellah Ajji

**Affiliations:** Département de Génie Chimique, Polytechnique Montréal, Montréal, QC H3C 3A7, Canada; dariush.karimi-alavijeh@polymtl.ca (D.K.A.); bentolhoda.heli@polymtl.ca (B.H.)

**Keywords:** colorimetric detection, gas indicator, anthocyanins, freshness indicator, natural dye, meat spoilage, intelligent packaging

## Abstract

This study aimed to fabricate and characterize a novel colorimetric indicator designed to detect ammonia (NH_3_) and monitor meat freshness. The sensing platform was constructed using electrospun nanofibers made from polylactic acid (PLA), which were then impregnated with anthocyanins as a natural pH-sensitive dye, extracted from red cabbage. This research involved investigating the relationship between the various concentrations of anthocyanins and the colorimetric platform’s efficiency when exposed to ammonia vapor. Scanning electron microscope (SEM) results were used to examine the morphology and structure of the nanofiber mats before and after the dip-coating process. The study also delved into the selectivity of the indicator when exposed to various volatile organic compounds (VOCs) and their stability under extreme humidity levels. Furthermore, the platform’s sensitivity was evaluated as it encountered ammonia (NH_3_) in concentrations ranging from 1 to 100 ppm, with varying dye concentrations. The developed indicator demonstrated an exceptional detection limit of 1 ppm of MH_3_ within just 30 min, making it highly sensitive to subtle changes in gas concentration. The indicator proved effective in assessing meat freshness by detecting spoilage levels in beef over time. It reliably identified spoilage after 10 h and 7 days, corresponding to bacterial growth thresholds (10^7^ CFU/mL), both at room temperature and in refrigerated environments, respectively. With its simple visual detection mechanism, the platform offered a straightforward and user-friendly solution for consumers and industry professionals alike to monitor packaged beef freshness, enhancing food safety and quality assurance.

## 1. Introduction

Microorganisms, present in small populations, play a significant role in the natural processes of living, multiplying, and metabolizing various substances found in food [1]. Normally, these compounds are present in minimal concentrations in biologically active tissues. However, improper processing and inadequate storage conditions can lead to premature food spoilage, as certain types of microorganisms such as *pseudomonas* and *lactobacillus* [2] thrive in uncontrolled environments due to the favorable conditions found therein. These microorganisms produce significant quantities of off-flavor compounds like carbon dioxide, volatile organic compounds (VOCs), and biogenic amines (BAs), which act as key markers for food spoilage detection. Notably, the total amount of volatile basic nitrogen (TVB-N) is the most reliable indicator of spoilage in meat products, as it is a major component of these breakdown gases [3,4]. Compounds like TMA, DMA, primary, secondary, and tertiary amines, and ammonia collectively contribute to the total volatile basic nitrogen (TVB-N) content, which increases as protein-based foods undergo putrefaction and amino acid deamination. In large quantities, these substances have been identified as major causative agents of food-borne diseases, including intoxications [5]. Therefore, it is crucial to monitor the presence of these toxins to assess the freshness of protein-based food products and determine if they have undergone spoilage.

Smart/intelligent packaging has created the possibility of monitoring the freshness of food products through detection and analyses. Intelligent packaging is a system of sensing, detecting, and recording any changes inside a food package [6].

The presence of amines and off-flavor compounds can significantly impact the pH of the surrounding environment, primarily due to their alkaline properties. This characteristic provides an opportunity to detect and track these compounds by monitoring pH levels. In recent developments, pH colorimetric indicators have been devised to be directly introduced into the headspace of food packaging [7]. By employing these indicators, qualitative or semi-quantitative information about the presence of microorganisms in the food can be obtained, offering valuable insights into its quality through visual colorimetric changes [8]. Consequently, in recent years, there has been an increasing interest in the development of a colorimetric sensing platform for identifying freshness in meat products, as they are inexpensive, uncomplicated, highly sensitive, and selective [9,10].

To achieve this, molecules capable of exhibiting distinct colors based on the pH of the environment have been extensively studied and utilized as essential components of these indicators. Preference has been given to naturally sourced molecules like anthocyanin over synthetic dyes such as cresol red, methyl red, bromocresol purple, phenol red, etc., as it offers low toxicity, high biocompatibility, and sustainability [11].

Anthocyanins are a natural source of pH-sensitive pigments found in various vegetables and fruits, such as grapes, berries, red cabbage, plums, etc. [12]. The pH-dependent color changes from pinkish in an acidic environment to bluish purple in basic surroundings [13], as displayed by anthocyanins, making it a valuable natural indicator for assessing the acidity or alkalinity of a given environment.

The unique behavior of anthocyanin in various environments with different pH levels has paved the way for its application in the development of colorimetric freshness indicators for food packaging purposes. Whether used alone or in combination with other pH-sensitive dyes, anthocyanin enables the detection of amines and provides information about the condition of the food. To achieve this, solid composite structures based on polymers, such as films, hydrogels, nanofibers mats, and other applicable forms, have been specifically engineered to be integrated into the headspace of food packages [7,14]. Of all these, a nanoporous platform fabricated with electro-spinning technology has generated a lot of attention due to its high surface-area-to-volume ratio [15]. Despite these advancements, challenges persist when creating a feasible and highly effective system for colorimetric indicators in food packaging. Some indicators fail to exhibit easily detectable color changes, while others lack a direct correlation between the visual color change and the actual condition of the food or the concentration of amines at which the change occurs. Additionally, certain indicators may not display a sufficiently visible color change in response to specific concentrations of amines. Previous studies have also overlooked crucial aspects, including the evaluation of pH-induced color changes in buffer solutions, testing with realistic NH_3_ concentrations, and specific off-flavor compounds responsible for microbial food spoilage.

Existing methods for detecting various target molecules in food products often face limitations such as limited sensitivity, specificity, and reliability, particularly when dealing with complex matrices and trace-level analytes. Additionally, many conventional techniques require extensive sample preparation, specialized equipment, and trained personnel, making them costly and time-consuming. Furthermore, some methods may lack versatility and struggle to detect multiple contaminants simultaneously. Overall, these limitations underscore the need for innovative approaches that offer enhanced sensitivity, specificity, simplicity, and cost-effectiveness for comprehensive food safety analysis [16,17,18,19].

These limitations may lead to misleading information, as the food can spoil before the indicator undergoes a color change, either because the change occurs at amine concentrations higher than the spoilage limit or due to a mismatch in the indicator’s response time. Therefore, it is imperative to develop colorimetric indicators with enhanced sensitivity and real-time monitoring capabilities that can accurately indicate the threshold beyond which the food is unsuitable for consumption. Overcoming these challenges will enable the creation of reliable, safe, and efficient colorimetric indicators for food packaging, ensuring food safety and quality assessment [20]. To overcome the existing challenges, it is crucial to explore materials with highly available active surface areas that can efficiently interact with amine compounds and undergo distinct color changes at defined concentrations. Porous materials, specifically those with a high active surface area, hold promise in facilitating this efficient interaction, leading to faster response times and increased sensitivity, compared to films made from the same materials.

Interestingly, despite the use of highly porous nanofibers in various fields such as filtration and biomedicine [7,21], their potential as colorimetric indicators integrated with natural-based dye for real-time meat spoilage has received limited attention so far. Table 1 displays a selection of the studies conducted in this field. Thus, this unexplored avenue presents an opportunity to harness the benefits of fibers in developing highly effective and sensitive indicators for food spoilage detection.

Various types of materials have been used as substrates, such as filter paper, film, sol-gel nanoporous, bacterial cellulose, and electrospun nanofiber mats. Of all these, a nanoporous platform fabricated with electrospinning has generated lots of attention due to its high surface-area-to-volume ratio [21]. By focusing on materials with a high surface-area-to-volume ratio, exploring sensitivity, and considering the usage of naturally extracted dyes, researchers can advance the development of colorimetric indicators for rapid food spoilage detection [9]. In this study, the developed colorimetric indicator, composed of electrospun nanofibers impregnated with anthocyanins, demonstrated high sensitivity and selectivity toward NH_3_, with a purplish-blue coloration detectable visually, even at 1 ppm. As a state-of-the-art technique, the fabricated indicator was applied to the headspace of meat, allowing for the monitoring of food safety over time. This indicator offers a novel approach to evaluating protein-based food, providing a user-friendly, safe, and cost-effective solution.

## 2. Materials and Methods

### 2.1. Materials

Polylactic acid (PLA) with a molecular weight of 133,000 g/mol (INGEOTM Biopolymer 4032D) was supplied from Nature Works LLC (Blair, NE, USA). 2,2,2-Trifluoroethanol (TFE, ≥99.9%), ethanol (EtOH, ≥99.9%), ammonium hydroxide (ACS reagent, 28.0–30.0% NH3 basis), dimethylamine (DMA, ≥99%), and trimethylamine (TMA, ≥99%) were purchased from Sigma Aldrich, Canada, and plate count agar (OXOID, dehydrated, OXCM0325B) was purchased from Thermo Scientific TM (Montreal, QC, Canada). Red cabbage and beef were freshly purchased from the local market (Montréal, QC, Canada). All chemicals were received and used without any further purification.

### 2.2. Extraction of Anthocyanin

The extraction proceeded according to the Fuleki and Francis (1968) procedure, with some modifications [13]. Red cabbage leaves were washed and cut into small pieces. The extraction was carried out by adding 150 g of chopped leaves to 80 mL of ethanol, with the pH adjusted to 2 using HCl 1 M in an Erlenmeyer flask (100 mL), and the solution was stored in the fridge (at 4 °C) for 24 h. It has been reported that the stability of anthocyanin increases with a decrease in pH level [33]. The diffused anthocyanin into the liquid was separated from the red cabbage leaves through filter paper (Whatman^TM^, Maidstone, UK, NO. 1). The extraction obtained from filtration was centrifuged (Thermo Scientific™ Sorvall™ RC 6 Plus) at 10,000 rpm for 15 min to remove fine suspended particles. Finally, the pH of the obtained filtrate was adjusted to 2, using HCl 1 M. The concentration of the anthocyanins was calculated by the pH differential method, using the following equation [33]:

(1) $$C=\frac{A\times MW\times D_{f}}{\varepsilon }\times L$$

(2) $$A={\left(A_{530}-A_{700}\right)}_{pH1.0}-{\left(A_{530}-A_{700}\right)}_{pH4.5}$$

where C is pigment concentration (mg/mL) and A is the difference of anthocyanin absorbance at the assigned wavelength (350 and 700 nm) and adjusted pH (1 and 4.5), as described in Equation (2). MW is the molecular weight of anthocyanin (449 g/mol), $D_{f}$ represents the dilution factor, ε is the extinction coefficient (26,900 L/cm^2^ mol), and L is the path length (1 cm).

### 2.3. Preparing PLA Electrospun Nanofibers

PLA solution at a concentration of 15% (*w*/*v*) was prepared as follows: first, the PLA pellets were dried at 70 °C for 4 h under vacuum conditions. Next, 1.5 g of PLA pellets were dropped into 10 mL of TFE and mixed overnight, under conditions of stirring at room temperature (23 °C).

**Figure 1:**
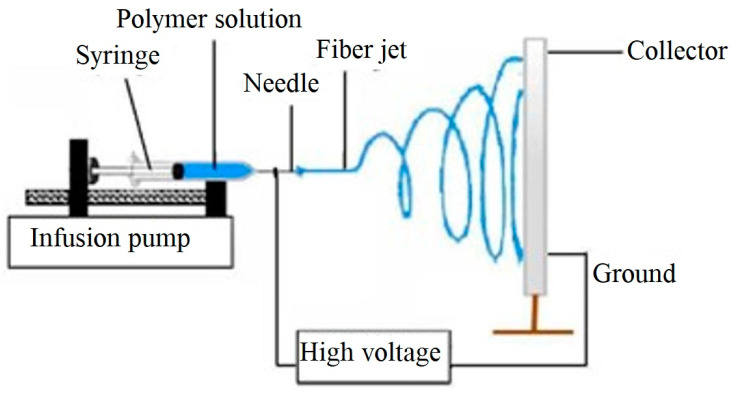
Schematic of electrospinning.

To obtain the electrospun nanofiber platform, the electrospinning set-up was used as shown in Figure 1. Accordingly, a 5 mL plastic syringe was filled with PLA solution and connected to a 23-gauge stainless steel needle. Then, it was placed in a syringe pump (Harvard Apparatus, PHD2000, Holliston, MA, USA) and the positive electrode of a power supply was clipped to the needle. The polymer solution was electrospun at a feed rate of 1 mL/h with a tip-to-collector distance of 15 cm. Through applying a voltage of 20 kV provided by a high-voltage power supply (ES60P-5W Gamma High Voltage Research Inc, Ormond Beach, FL, USA) nonwoven nanofibers were fabricated on a grounded stationary collector (connected to the negative electrode of the power supply) and covered with aluminum foil. All the electrospinning experiments were carried out at room temperature (23 °C) and at a relative humidity of around 50%.

### 2.4. Fabrication of the Indicator

The dip-coating method was used to immobilize the extracted anthocyanin from red cabbage on a PLA electrospun nanofiber. In brief, a central piece of PLA substrate, cut to a size of 5 × 5 cm^2^ and an average thickness of 0.4 mm, was immersed into 20 mL of the dye solution and placed on a shaker (Standard Analog 1000 Orbital Shaker, 120 V, TALBOYS) for 24 h. To optimize the concentration of anthocyanin for the sensing platform, dye solutions with various concentrations were prepared (the initial solution of extracted dye was diluted 10 and 100 times by ethanol). The immobilization of the dyes is attributed to the physical adsorption of the anthocyanin pigments into the nanoporous platforms. The drying steps were carried out first; we positioned the mats on a handmade clotheshorse for 48 h to evaporate the residual solvent on the mats, then placed them under the laboratory fume hood at room temperature to ensure that all the solvents evaporated.

### 2.5. Characterization of the Electrospun Nanofiber Mats

The morphology of the resulting electrospun nanofibers was analyzed using a scanning electron microscope (SEM, TM3030PLUS, HITACHI, Chiyoda City, Tokyo) operating at 10–15 kV. The fiber diameter was also evaluated using the open-source LAB ORIGIN software (2015, version 92E, copyright 1991–2014 OriginLab Corporation, Northampton, MA, USA). The weight and thickness of the sheets were measured using a balance (ML 3002E, Mettler Toledo, Greifensee, Switzerland) and a micrometer (Mitutoyo 547-401 ABSOLUTE Digimatic Thickness Gauge, Kanagawa, Japan), respectively. The porosity of the nanofibrous mats was calculated using two different methods: apparent density measurements [21] and liquid (ethanol) intrusion [34]. For the apparent density method, the density of the electrospun mat (ρ) was calculated by obtaining the weight and thickness of a circular area of 6 mm in diameter, which was precisely punched. As reported in the datasheet, the initial density value ${(\rho }_{0}$) of PLA was 1.25 g·cm^−3^ [35]. Then, the porosity (P%) was calculated using the following equation:

(3) $$P\%=\frac{\rho_{0}-\rho }{\rho_{0}}\times 100$$

The porosity of mats was also determined using a liquid (ethanol) intrusion method. In such a method, dry mats with a size of 5 × 5 cm^2^ were weighted and immersed in pure EtOH overnight. The mats were then gently wiped to remove surplus EtOH and weighed again. The porosity was defined as the volume of EtOH entrapped in the pores divided by the total volume of wet mats. The porosity calculated in this method was comparable with the one estimated with Equation (3). The calculated porosities of the developed substrate are presented in Table 2 in the results and discussion section.

### 2.6. Evaluating the Performance of the Indicator

The colorimetric gas-sensing experiments were carried out using a homemade detection setup consisting of a sealable glass container with a total volume of 900 mL placed on a scanner (Epson Canada Ltd., Toronto, ON, Canada, Perfection V550).

The developed indicator (a circular shape with a 6 mm diameter assembled inside aluminum foil and fixed inside of the container) was exposed to the evaporated NH_3_ at different concentrations varying from 1 to 100 ppm, which was provided through the simultaneous evaporation of ammonium hydroxide solution with various concentrations at room temperature. The concentration of NH_3_ was calculated using the following equation [33]:

(4) $${NH}_{3}\;concentration\;(ppm)=\frac{M\times T}{V\times MW\times 12.178}$$

where M represents the weight of NH_3_ (mg), T is the temperature (K), V represents the volume of the container (900 mL), and MW is the molecular weight of NH_3_ (17 g/mol).

Subsequently, digital images of the indicator placed inside of the sealed container were captured by a scanner during its exposure to NH_3_ over a period of 60 min in a darkened surrounding area.

To verify the selectivity and specificity of the developed colorimetric indicator, volatile organic compounds such as dimethylformamide, x-xylene, formaldehyde, ethanol, tetrahydrofuran, methanol, dichloromethane, and acetone, and volatile nitrogen-based amines, such as dimethylamine, trimethylamine, and ammonium hydroxide, were exposed to the indicators. As such, 100 µL of the specific solution was dropped to the sealed container, and the volatile compound was supplied by its evaporation at room temperature. The color change of the indicator was recorded during the 60 min exposure period, as mentioned before.

The scanned image of the initial and exposed indicator platforms was imported into MATLAB (version 9.5.0) for quantitative analysis. The RGB (red, green, and blue) difference model (ΔRGB) was used to read the color information and differences before and after exposure to NH_3_ and other compounds. The RGB model in MATLAB decomposes the color of each image into the components red (R), green (G), and blue (B) and quantitively shows them in a three-dimensional matrix. By calculating the difference between these components in each image, the performance of the sensing platform can be evaluated. The following equation represents the calculation of this model, wherein the subscript 0 corresponds to the initial image:

(5) $$\Delta \mathrm{RGB}=\sqrt{{\left(R-R_{0}\right)}^{2}+{\left(G-G_{0}\right)}^{2}+{\left(B-B_{0}\right)}^{2}}$$

### 2.7. Sensitivity of the Indicator for Detecting Meat Spoilage

As a proof-of-concept, the developed indicator was applied to examine the freshness of real packed meat through its sensitivity to produced gases during the spoilage of meat. This sensitivity was also validated by comparing it with the microbial growth within meat samples. As such, 100 g of fresh beef was cut and prepared under sterile conditions and then transferred to a sealable container, with the indicator (a circular shape with a 6 mm diameter assembled inside aluminum foil), horizontally attached to the sealed container. The whole sensing setup was assembled onto the scanner, which was plugged into the computer system to simultaneously scan and process the sensing behavior of the colorimetric indicator. The indicator performance was evaluated for both meat samples stored at room temperature and 4 °C.

### 2.8. Bacterial Analysis

To analyze the presence of bacteria on a piece of meat, one hundred grams of fresh beef was cut under sterile conditions. Then, the prepared samples were stored at room temperature or at 4 °C, before their bacterial growth was evaluated, according to the indicator scanning time. As such, the meat samples, at the appropriate time, were mixed with 25 mL of PBS (phosphate buffer saline) (0.01 M, pH 7.4). Then, the mixture was shaken and homogenized in a vortex (Scientific Industries, Vortex-Genie 2, Biotech Inc., Montréal, QC, Canada) for 5 min. Next, 0.1 mL of the prepared sample was serially diluted down six times. Finally, 0.01 mL of each dilution was spread onto the surface of an agar plate to determine the TVC (total viable count). The agar plate was kept in the incubator (Economy Incubators IB-11E, 150 L, Biotech Inc., Montréal, QC, Canada) for 18 h at 37 °C to grow the bacterial colonies before counting [34].

## 3. Results and Discussion

### 3.1. Characterization of Nanofiber Mats

**Figure 2:**
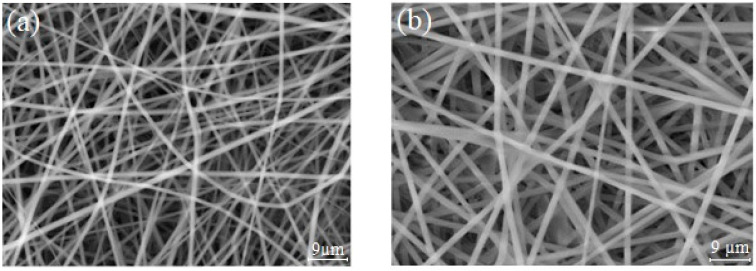
SEM images of (**a**) electrospun PLA nanofibers, (**b**) the anthocyanin-impregnated PLA sample.

SEM images of electrospun PLA nanofibers before and after dye introduction are presented in Figure 2. The images show the morphology of the pristine mats, comprising randomly arranged nanofibers. Before dip-coating (Figure 2a), an average nanofiber diameter of 0.9 ± 0.15 µm was determined, indicating the initial structure of the nanofiber mats. Following dye-coating (Figure 2b), the overall morphology of the PLA nanofiber mats remained unaltered, with negligible changes observed in the average nanofiber diameter.

**Table 2:** Comparison of the properties of PLA nanofiber, before and after dye-coating.

Sample	Fiber Diameter (µm)	Thickness (µm)	Density (g/cm^3^)	Porosity %
PLA nanofiber	± 0.15	400 ± 56	0.175 ± 0.01	86 ± 1
PLA–dye nanofiber	1.02 ± 0.15	400 ± 70	0.183 ± 0.01	84 ± 1

However, upon closer examination, a marginal reduction in the porosity of the dye-impregnated nanofiber samples was noted. This reduction in porosity can be attributed to the increased mat weight resulting from the dye-coating process. Despite this slight alteration, a comprehensive comparative analysis of the platform’s properties, outlined in Table 2, confirmed that the coating procedure had minimal influence on the inherent qualities of the nanofiber mats. This outcome emphasizes the efficacy of the simple dip-coating technique in fabricating a uniform nanoporous layer impregnated with anthocyanin. It also demonstrates its homogeneity and suitability for the intended application in colorimetric indicators.

It is worth mentioning that filter paper and bacterial cellulose were compared with PLA electrospun nanofibers as substrates (results were not presented here). The results indicated that PLA nanofibers provided a more precise response due to their high aspect ratio and porosity. Although bacterial cellulose had fast responses, its transparency led to imprecise delta RGB values. Thus, PLA electrospun nanofibers were the optimal substrate, showing acceptable strength during testing.

### 3.2. Selectivity of the Indicator

**Figure 3:**
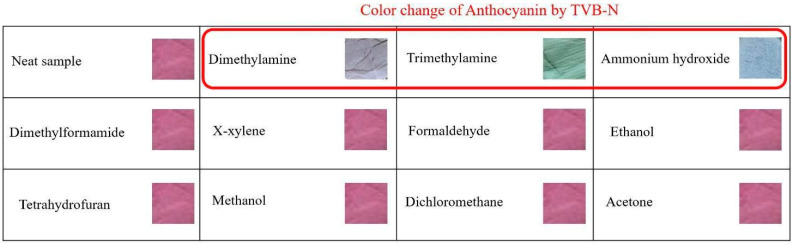
Selectivity of the indicator toward different VOCs.

The assessment of the indicator selectivity and sensitivity was carried out by gauging the ΔRGB (color change). The indicators impregnated with anthocyanin concentration of 570 µg/mL were subjected to different volatile organic compounds (VOCs) produced by 100 µL of its solutionfor 60 min. As shown in Figure 3, notable chromatic transformations were evident upon exposure of the indicator to DMA, TMA, and NH_3_. Specifically, the indicator altered from pink to violet, green, and blue upon interaction with DMA, TMA, and NH_3_, respectively. Conversely, the indicator’s color remained unaltered when confronted with various alternative organic compounds. This result reveals the selectivity of the prepared indicator toward amine-based compounds.

### 3.3. Stability of Indicator toward Humidity

**Figure 4:**
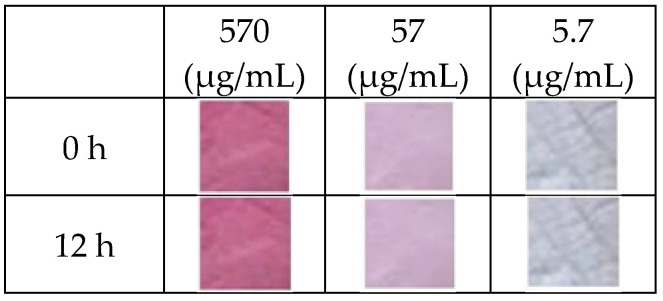
Optical images of indicators impregnated with various concentrations of anthocyanin before and after exposure to moisture (100% relative humidity).

The indicator’s robustness in the presence of moisture stands as a critical feature that may shape its potential applications. In assessing the indicator’s interaction with the moisture, specimens were subjected to a nitrogen gas environment saturated with 100% humidity for 12 h. The stability of indicators impregnated with various concentrations of anthocyanin dye (570, 75, and 7.5 µm/mL) was compared. As seen in Figure 4, the indicator’s color did not change before or after being exposed to moisture, implying that water had no effect on the indicator’s color.

### 3.4. Examining the Sensitivity of the Colorimetric Indicator toward Ammonia

To optimize the efficacy of anthocyanin as a pH indicator dye, an assessment of various dye concentrations was explored, followed by systematic testing to confirm the highest sensitivity and colorimetric performance. Subsequently, the indicator’s response to varying NH_3_ concentrations from 1 to 100 ppm and to various exposure times was measured, to uncover its efficacy.

**Figure 5:**
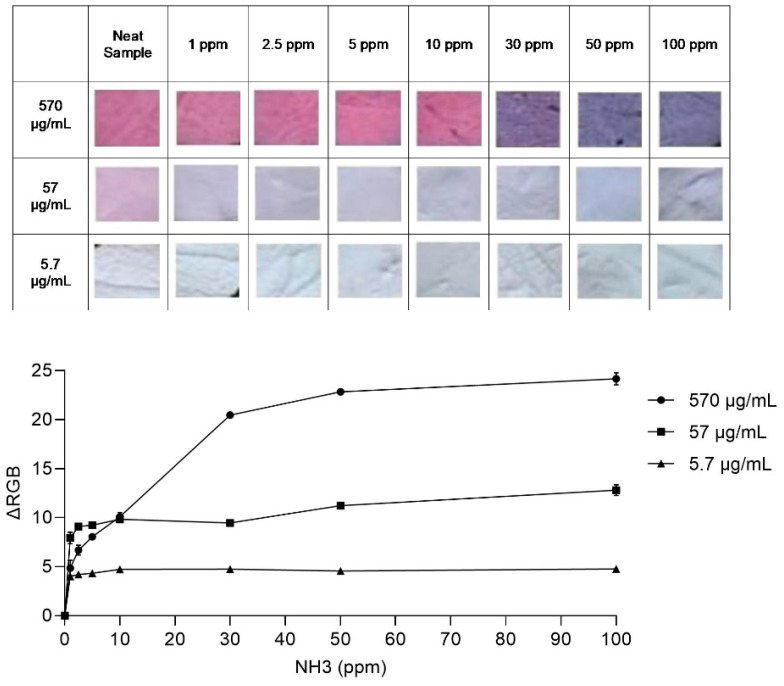
The response of indicators with varying anthocyanin concentrations (570 µg/mL, 57 µg/mL, and 5.7 µg/mL) after 60 min exposure to ammonia, including optical images of indicators captured at different NH_3_ concentrations ranging from 1 to 100 ppm, and the corresponding calibration curves between ammonia concentrations and the changes in color of the indicators (ΔRGB).

All the indicators initially exhibited a pink hue, despite varying degrees of intensity in their unexposed state. Upon exposure to NH_3_, an acid–base reaction prompted the deprotonation of the dye molecules, gradually transitioning their initial color to a distinct blue shade [35]. The alteration in dye concentration directly impacted the indicator’s reactivity—lower dye concentrations reduced the available reactive sites. Consequently, a reduced concentration of NH_3_ gas (as low as 1 ppm) was sufficient to induce a reaction with the dye at a concentration of 5.7 µg/mL, leading to heightened sensitivity, as illustrated in Figure 5.

Nonetheless, diluting the dye also correlated with a decrease in the RGB value, potentially complicating the naked-eye perception of color changes. Given the semi-quantitative nature of human color detection, RGB analysis becomes imperative [36]. The colorimetric response (ΔRGB) to NH_3_ concentration was explored for indicators impregnated with varying dye concentrations following a 60 min NH_3_ exposure, as shown in Figure 5. The resulting calibration curve for NH_3_ detection, across different dye concentrations, displays a notable range of variation spanning from 1 to 100 ppm.

In Figure 5, it can be observed how different concentrations of the dye affected the behavior of the indicators in response to varying levels of ammonia. When the dye concentration was high (570 µg/mL), it reached a point where the color change leveled off or became saturated at around 30 ppm of ammonia. This meant that increasing the concentration of ammonia beyond this point did not cause a significant change in the color of the indicator. On the other hand, when the dye concentrations were lower (57 µg/mL and 5.7 µg/mL), the color change leveled off at much lower concentrations of ammonia, specifically within the 1 ppm range. This is remarkable because it indicates that, at such low concentrations, the color change is discernible to the naked eye without requiring specialized equipment for detection.

The steepness of these slopes reflects the sensitivity of the indicator to changes in ammonia concentration. When the slope is steeper, it means that even small changes in ammonia concentration caused significant changes in the color of the indicator. Thus, a steeper slope can be interpreted as an indication of the higher sensitivity of the indicator. By carefully adjusting the composition of the dye solution, it is possible to enhance the limit of detection of the indicator to below 1 ppm of ammonia. This demonstrates the potential for further refinement and optimization of the indicator’s sensitivity, which is crucial for detecting very low levels of ammonia accurately.

However, it is important to note that using a very low concentration of anthocyanin dye, such as 5.7 µg/mL, may present challenges in real-life applications. One such challenge is the difficulty in perceiving the color change visually due to its subtle nature at such low concentrations. Therefore, while striving for increased sensitivity, it is essential to ensure that the color change remains perceptible and practical for real-world use.

**Figure 6:**
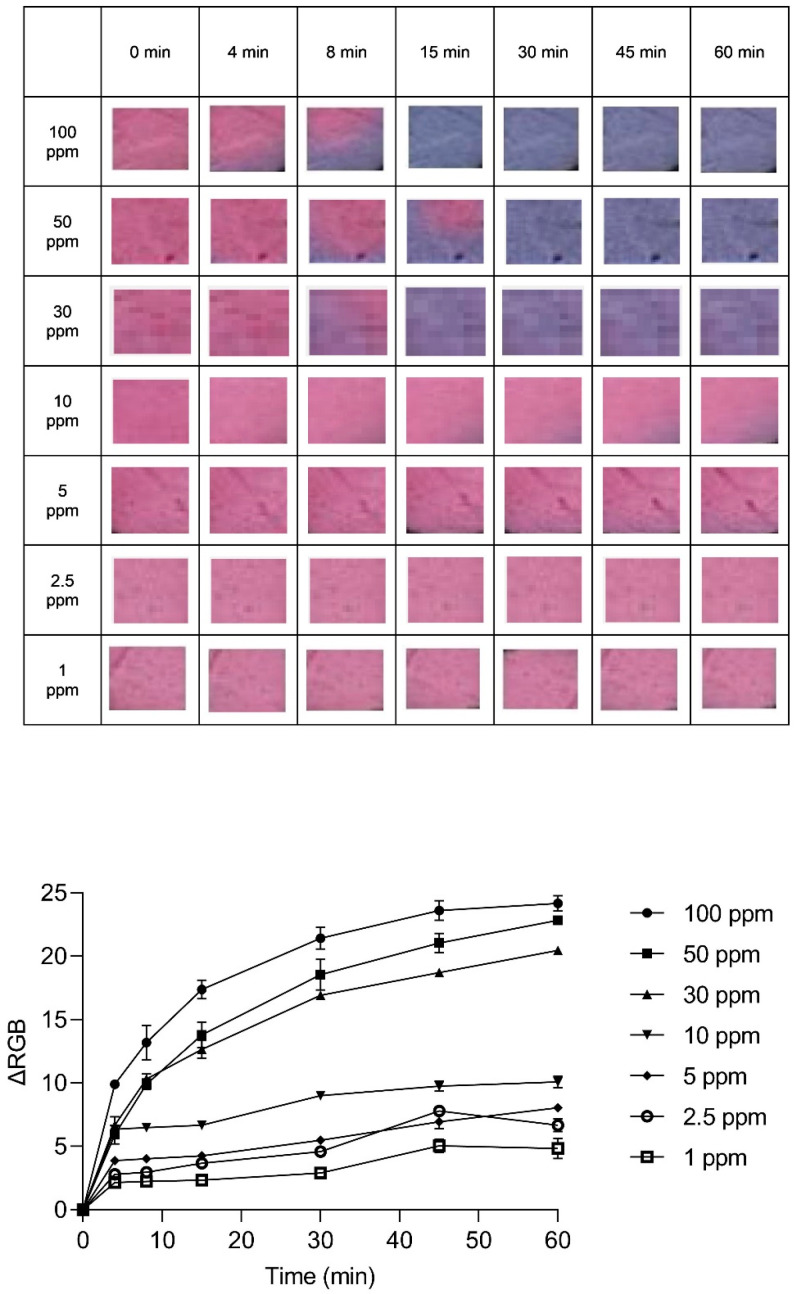
The variation in ΔRGB of the indicator with a dye concentration of 570 µg/mL, along with optical images of the indicator at various NH_3_ concentrations (ranging from 1 to 100 ppm) over 60 min.

**Figure 7:**
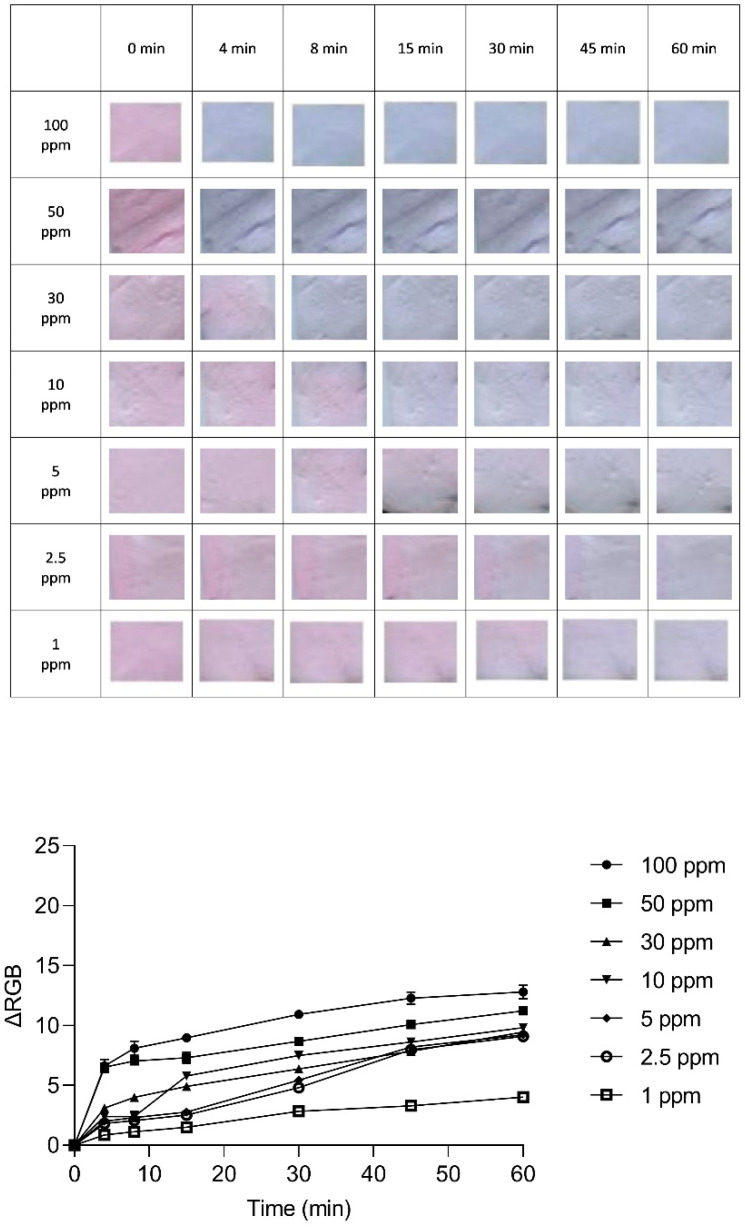
The variation in ΔRGB of the indicator with a dye concentration of 57 µg/mL, along with optical images of the indicator at various NH_3_ concentrations (ranging from 1 to 100 ppm) over 60 min.

**Figure 8:**
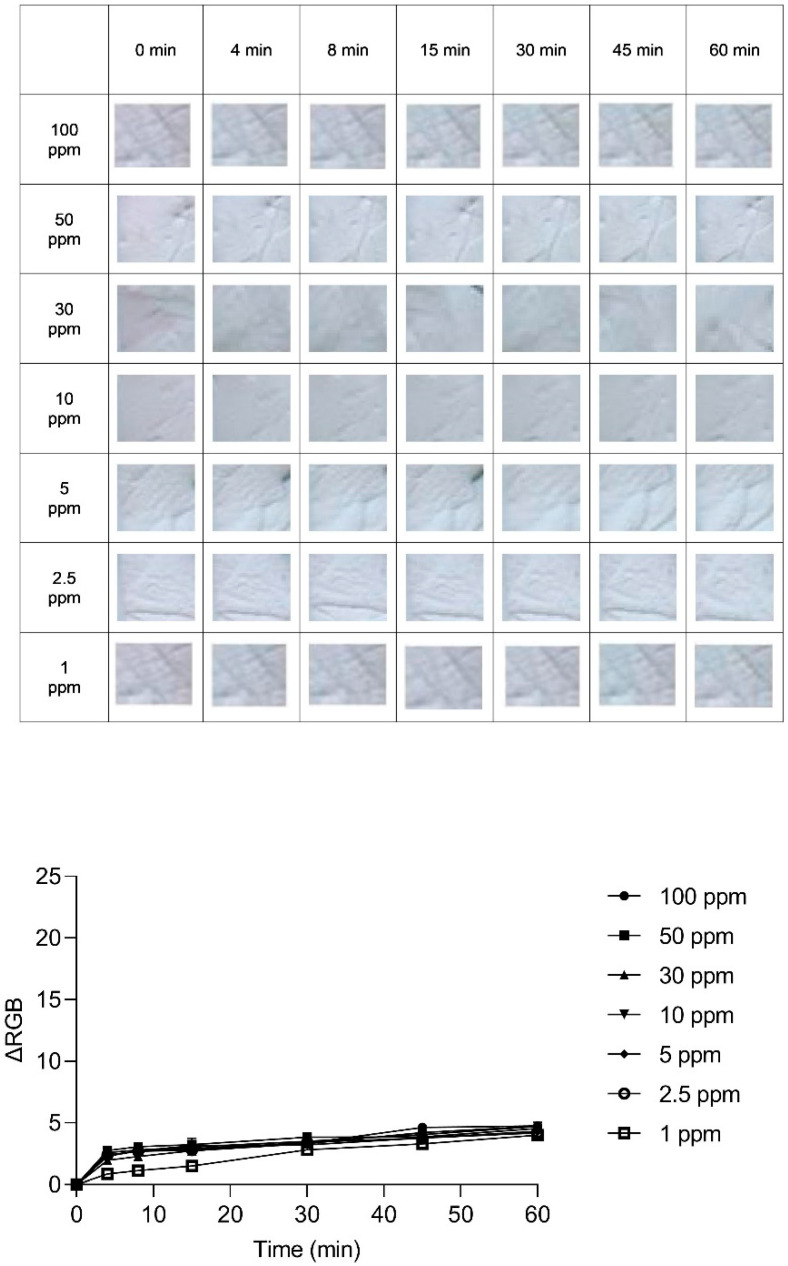
The variation in ΔRGB of the indicator with a dye concentration of 5.7 µg/mL, along with optical images of the indicator at various NH_3_ concentrations (ranging from 1 to 100 ppm) over 60 min.

An exploration into the kinetic behavior of the anthocyanin-impregnated PLA nanofiber platform was conducted to unveil the dynamics of its color transformation. The (ΔRGB) variations were meticulously observed over time across different dye concentrations, as illustrated in Figure 6, Figure 7 and Figure 8. These variations offer insights into the relationship between exposure duration and dye concentration.

The optical snapshots captured during a span of 60 mins’ exposure to NH_3_, illustrated in these figures, depict the progressive shift from pink to bluish-purple across the exposure timeframe. In the context of a dye concentration of 570 µg/mL, as illustrated in Figure 6, the color transition commenced within 8 min and reached saturation at around 15 min when exposed to 100 ppm NH_3_. Notably, minimal color alteration is discernible at concentrations below 10 ppm.

Turning our attention to Figure 7, with a dye concentration of 57 µg/mL, a swift and distinct color shift from pink to blue is evident even at the low NH_3_ exposure concentration of 1 ppm. Conversely, the dye concentration of 5.7 µg/mL in Figure 8 illustrates an insignificant color change, with the RGB distance residing within the range of visual error for the naked eye.

The deliberate deceleration in response time, particularly observable at low concentrations, suggests that the kinetics of ΔRGB alteration are inherently limited by the transfer rate of NH_3_ molecules. A similar notion was observed by Hoang et al., highlighting the influence of mass transfer on the kinetic color change of indicators [37].

Interestingly, the emergence of the blue color initially at the indicator’s periphery before diffusing across the entire surface could potentially be attributed to the direction of NH_3_ flow, underscoring a nuanced interplay between gas movement and color propagation.

In acidic and neutral conditions (pH = 3–7), anthocyanin takes on a reddish color, primarily due to the prevalence of its flavylium cation form. The flavylium cation has a positive charge delocalized over the oxygen and nitrogen atoms of the heterocyclic ring structure. This form appears red because it absorbs green light. However, in alkaline conditions (pH above 7), anthocyanins undergo structural changes and convert to a blue–green color, due to an open-chain structure called the carbinol pseudo-base. The carbinol pseudo-base form has an open ring structure with no delocalized charge, resulting in a green color in anthocyanin [23].

### 3.5. Colorimetric Sensing of Meat Spoilage

**Figure 9:**
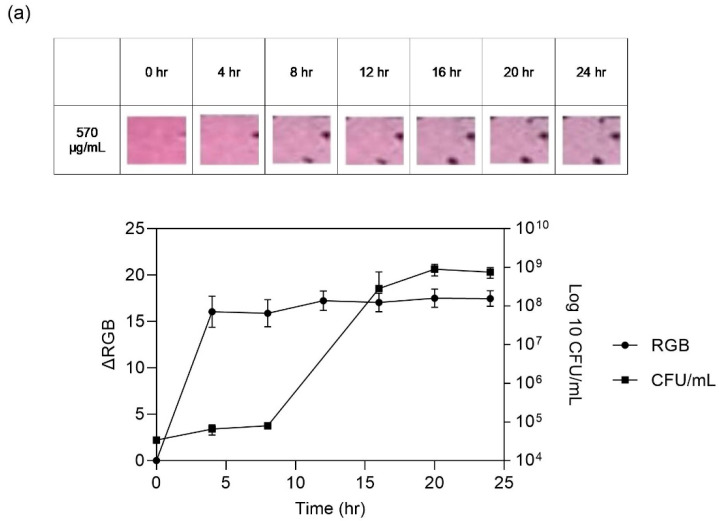

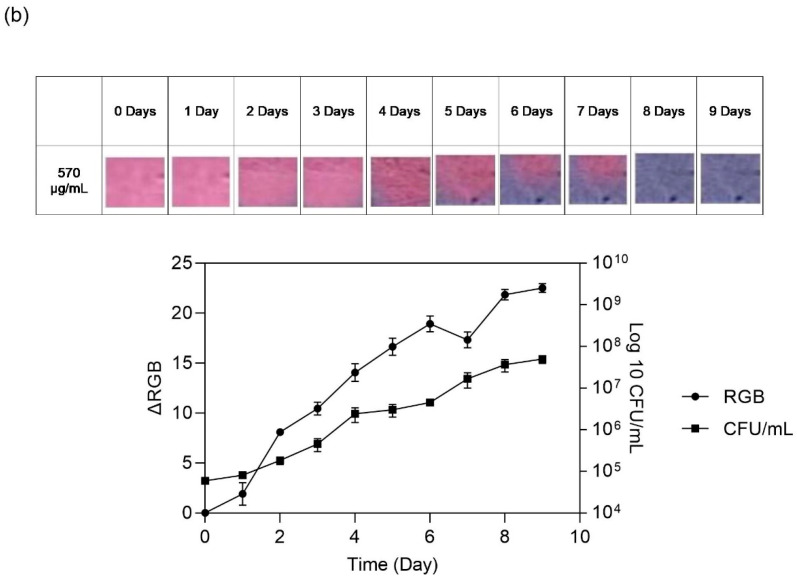
Colorimetric analysis, optical image, and bacterial growth during beef spoilage at (**a**) room temperature (23 °C) over 24 h and (**b**) 4 °C over 9 days.

The selectivity of the developed platform encompasses amine compounds, aligning with the typical threshold of 10^7^ CFU/mL established for fresh meat [38,39]. Leveraging the sensitivity exhibited by the colorimetric platform, a dye concentration of 570 µg/mL was selected for the assessment of meat spoilage. To ascertain the spoilage progression in beef, an investigation was conducted over a 24 h duration under ambient conditions (23 °C) and over 9 days in the refrigerator at 4 °C. The indicator’s response was employed as a gauge of meat spoilage. Figure 9 clearly demonstrates the findings, incorporating ∆RGB analysis, the optical appearance of the indicator, and subsequent bacterial growth evaluation, following exposure to 100 g of fresh beef. The alterations in the indicator’s color serve as a reliable approach of the presence of amine compounds, which emerge during meat spoilage due to bacterial growth. The expected outcome is that the color change becomes noticeable and reaches saturation before the meat is fully spoiled, as anticipated.

Bacterial duplication was further assessed using the Total Viable Count (TVC) method, tracking the growth trajectory of microorganisms while the meat was stored at both room temperature (23 °C) and refrigeration temperature (4 °C). Notably, the TVC revealed that bacterial spoilage crossed the threshold after 10 h at room temperature, which was extended to seven days under refrigeration conditions.

Concurrently, the colorimetric platform exhibited detectable changes in as little as 5 h at room temperature and similarly, seven days at refrigeration temperature, as evidenced by ΔRGB analysis. Importantly, this outcome demonstrated a robust correlation with bacterial growth trends. The indicator showed commendable sensitivity, displaying discernible color changes before full meat spoilage occurred. While the TVC progressively escalated from the threshold point, the color change intensity within the colorimetric indicator reached a saturation plateau. This convergence affirms the platform’s efficacy in capturing spoilage occurrence, ensuring that its color alteration properties offer a reliable and timely indicator of meat deterioration.

This research demonstrates the capability of a highly porous electrospun nanostructure for providing real-time colorimetric detection of NH_3_ and the continuous monitoring of meat freshness. In contrast, the majority of prior studies have presented colorimetric indicators containing natural dyes [23,38,39], which primarily assess the platform’s ability to change color in the presence of various volatiles, as well as real applications for beef, poultry, or seafood [40,41,42,43,44,45], without proper investigations of indicator sensitivity to volatile concentrations with respect to dye concentration as well as color-changing kinetics [46]. Interestingly, our work provides a comprehensive assessment of the system’s overall performance, including the responsiveness, sensitivity, and selectivity of PLA dip-coated anthocyanin at various concentrations and under various experimental conditions.

## 4. Conclusions

The research findings hold significant implications for the future of food safety analysis. The development of a sensitive colorimetric indicator using pH-sensitive dye derived from a natural source, such as red cabbage-extracted anthocyanin, opens new avenues for rapid and reliable detection methods. The remarkable stability, high sensitivity, and swift detection times achieved in this study pave the way for advancements in food safety monitoring. With rapid detection for low concentrations of NH_3_, coupled with impressive selectivity, this indicator offers a powerful tool for detecting spoilage in food products. Moreover, the indicator’s ability to detect VOCs at concentrations as low as 1 ppm further enhances its utility in food safety analysis. The straightforward colorimetric platform presented in this research holds promise for various applications, particularly within the food packaging industry, where it can play a vital role in ensuring product freshness and safety. Future developments based on these findings could lead to the widespread adoption of cost-effective and environmentally friendly methods for food safety analysis, benefiting both consumers and producers alike.

## Figures and Tables

**Table 1:** List of colorimetric indicators developed with natural dyes using various substrates.

Detector Agent	Substrate	Target Detection	Ref.
curcumin	k-carrageenan film	pork and shrimp	[22]
curcumin	gum/polyvinyl alcohol film	shrimp	[23]
anthocyanins	filter paper	shrimp	[24]
anthocyanins	chitosan film	fish and pork	[25]
anthocyanins	pectin/chitosan composite	beef	[26]
anthocyanins	sensory pad	chicken	[27]
anthocyanins	chitosan/PVA * films	time-temperature indicator	[19]
anthocyanins	bacterial-cellulose nanofibers	pH indicator	[7]
anthocyanins	chitosan film	pH indicator	[28]
anthocyanins	zein electrospun mat	pH indicator	[29]
anthocyanins	cassava starch	chilled pork	[14]
anthocyanins	gum composite film	chicken	[30]
anthocyanins	sol–gel film	pH indicator	[31]
anthocyanins	cassava starch film	pH indicator	[32]
anthocyanins	chitosan film	time-temperature indicator	[18]

* Polyvinyl alcohol (PVA).

## Data Availability

No new data were created or analyzed in this study. Data sharing is not applicable to this article.
